# Identification and Economic Evaluation of Differentiated Thyroid Cancer Care Consumption Patterns Using Sequence Analysis

**DOI:** 10.3389/ijph.2024.1606664

**Published:** 2024-04-19

**Authors:** Romain Demeulemeester, Pascale Grosclaude, Solange Grunenwald, Philippe Saint-Pierre, Nicolas Savy, Nadège Costa

**Affiliations:** ^1^Unité d’Evaluation Médico-Economique, Centre Hospitalier Universitaire de Toulouse, Toulouse, France; ^2^ Unité Mixte de Recherche1295 Centre d’Epidémiologie et de Recherche en Santé des Populations (CERPOP), Toulouse, France; ^3^Service d’Endocrinologie, Centre Hospitalier Universitaire de Toulouse, Toulouse, France; ^4^ Unité Mixte de Recherche5219 Institut de Mathématiques de Toulouse (IMT), Toulouse, France

**Keywords:** differentiated thyroid cancer, costs, care consumption patterns, optimal matching, clustering

## Abstract

**Objectives:** This study aims to assess the impact of care consumption patterns and individual characteristics on the cost of treating differentiated thyroid carcinoma (DTC), in France, with a specific emphasis on socioeconomic position.

**Methods:** The methodology involved a net cost approach utilising cases from the EVATHYR cohort and controls from the French National Health Insurance database. Care consumption patterns were created using Optimal Matching and clustering techniques. The individual characteristics influence on patterns was assessed using multinomial logistic regression. The individual characteristics and patterns influence on care costs was assessed using generalised estimating equations.

**Results:** The findings revealed an average cost of €13,753 per patient during the initial 3 years. Regression models suggested the main predictors of high DTC specific care consumption tended to include having a high risk of cancer recurrence (OR = 4.97), being a woman (OR = 2.00), and experiencing socio-economic deprivation (OR = 1.26), though not reaching statistical significance. Finally, high DTC-specific care consumers also incurred higher general care costs (RR = 1.35).

**Conclusion:** The study underscores the increased costs of managing DTC, shaped by consumption habits and socioeconomic position, emphasising the need for more nuanced DTC management strategies.

## Introduction

The incidence of differentiated thyroid carcinoma (DTC) has increased globally over the last 30 years. This has mainly been due to an increase in small papillary thyroid cancers, most of which have a good prognosis. Indeed, the improvement of diagnostic tools allowing the detection of cancers at an increasingly early stage and the evolution of surgical practices, with notably an increase in the number of total thyroidectomies performed, have progressively led to an increase in the number of diagnoses of small and very good prognosis cancers. A recent publication estimated that 70%–80% of thyroid cancer cases diagnosed between 2003 and 2007 were due to changes in diagnostic methods in several developed countries such as the United States, Italy, France, and Australia [1].

Although new options have become available in recent years, the main therapeutic strategy for treating DTC remains full or partial surgical removal of the thyroid, which may be combined with subsequent radioiodine therapy (RAI) [2]. The diagnosis of thyroid cancer is usually postoperative, but whatever the procedure performed, subsequent management should be tailored according to prognosis. To this end, several guidelines have been published such as the American Thyroid Association (ATA)’s Management Guidelines for Adult Patients with Thyroid Nodules and Differentiated Thyroid Cancer [3], giving recommendations about initial intervention and follow-up strategy depending on the initial risk of relapse.

Thus, as DTC is generally associated with a favourable prognosis, the increase in its diagnosis and treatment inevitably leads to an increase in the consumption of medical resources and corresponding costs, as shown by several studies. Indeed, Boltz et al. estimated the average first year cost per patient at $17,000 in the US between 1995 and 2005 while Finnerty et al. estimated it at about $14,000 between 2009 and 2013 [4, 5]. In France, a recent study by Li et al. estimated the mean cost per patient of thyroid cancer management between 2012 and 2016 at €7,351 for the first 2 years of care [6]. However, these studies did not focus on the overall cost of thyroid cancer but only on the management costs. In addition, the impact of socioeconomic position on thyroid cancer has been well established, with studies demonstrating that individuals from disadvantaged socioeconomic backgrounds are generally diagnosed at a more advanced stage of the disease, hence potentially affecting treatment options and clinical outcomes [7, 8]. To the best of our knowledge, no study has yet evaluated the cost of DTC in France nor the impact of different potential care consumption patterns and of the socio-economic position on these costs. We hypothesise that the management cost of differentiated thyroid carcinoma (DTC) in France is influenced by care consumption patterns and individual characteristics such as socio-economic position. In particular, based on existing knowledge, we anticipate that individuals from socioeconomically disadvantaged backgrounds will consume DTC specific care at a higher frequency and incur higher overall healthcare costs.

The main objective of this study is, therefore, to evaluate the impact of care consumption patterns as well as individual characteristics on the cost of DTC. To this end, it is therefore necessary to first evaluate the cost of DTC. Subsequently, the identification of care consumption patterns is formulated as an unsupervised classification of the state sequence problem, involving the computation of pairwise dissimilarity measures using Optimal Matching Analysis [9–11].

## Methods

### Material

Two data sources were used in combination to address the objectives of this study.

The EVATHYR cohort database, collected by the Midi-Pyrénées regional oncology network Oncomip, provided crucial information for understanding initial DTC management. All patients over the age of 18 diagnosed with a good DTC prognosis in 2014 in any administrative region of the Midi-Pyrénées region whose medical file was discussed in Multidisciplinary Team Meetings and registered in shared regional oncology software were included. Patients whose surgical management was carried out outside Midi-Pyrénées or who had another morphological type were excluded. The histopathology reports from biopsies and surgical specimens, biology reports, screenings, operative records, and complementary treatments proposed to patients are recorded, encoded, and anonymised in this database.

On the other hand, the SNIIRAM-PMSI database of the French National Health Insurance (FNHI) contains comprehensive information on healthcare consumption for 97% of the French population. This extensive and exhaustive database has been widely used for epidemiological research and health economic purposes. In this study, data related to DTC patients’ costs and healthcare consumption were extracted from the SNIIRAM-PMSI databases and provided invaluable insight into the costs of care and healthcare utilisation.

The EVATHYR cohort initially contained 361 DTC patients, 282 of whom were found in the SNIIRAM-PMSI databases. Of those 282 patients, 254 received healthcare services recorded in the SNIIRAM-PMSI databases throughout the study period. These elements were presented in the flowchart in Supplementary Appendix S1. In addition, a comparison between the baseline characteristics of the included and excluded populations is presented in Supplementary Appendix S2.

### Method

#### Mining the Outcomes of Interest

DTC management costs were assessed using a net cost method through a case-control study. For each case included in the EVATHYR cohort, the data of three controls who had never been treated for DTC were extracted from the SNIIRAM-PMSI by matching on age, gender, and administrative region of residence.

The evaluation was conducted from an FNHI perspective. The costs considered were direct medical costs (hospital stays, ambulatory care, drugs) and non-medical costs (transportation) as well as costs related to daily allowances given by the FNHI due to absence from the workplace. The various resources consumed are already valued in the SNIIRAM-PMSI databases. More specifically, all costs were obtained by applying the reimbursement rate of each consumed resource to its base tariff, then subtracting the number of deductibles and adding the number of supplements. All costs found by our research were expressed in 2024-euros while costs found in other studies were projected in 2024-euros using the methodology described by Turner et al. [12].

First, the overall average net cost of care, defined as the differential between the average cost of cases and matched controls, was described, including the detailed cost of services listed in Supplementary Appendix S3. The cost of cases and controls was evaluated on a quarterly basis, with a breakdown by expenditure item. This comprehensive evaluation provided a detailed understanding of the overall cost of care.

Second, the focus shifted to the gross cost associated with the general healthcare consumption of cases, which included all healthcare services and treatments used by these patients outside of the specific services (described in Supplementary Appendix S3) that were used to define the care consumption patterns. The impact of care consumption patterns and individual characteristics on the cost of general healthcare services was studied. The aim was to determine whether the degree of consumption of specific services for the management of DTC was associated with higher or lower consumption of general healthcare services.

The different care consumption patterns were then identified by applying the methodology of Optimal Matching Analysis [9–11] to the SNIIRAM-PMSI data. Each patient’s pattern was defined as an ordered sequence of monthly states over a 3-year time horizon. The date of each patient’s first surgery was taken as the index date of each follow-up. The different states were based on the monthly sum for the use of different services specific to the management of DTC, including hospital stays for interventions on the thyroid or parathyroid glands, RAI, scintigraphies, cervical ultrasounds, dosages of thyroglobulin, anti-thyroglobulin antibodies and thyroid-stimulating hormone, and consultations with an endocrinologist. As surgical interventions can lead to recurrent paralysis requiring speech therapy, consultations with an ear-nose-throat or speech specialist were also counted. The details of the services sought and the codes used to identify them in the SNIIRAM-PMSI databases, which were derived from the Common Classification of Medical Acts and the French National Table of Biology, are listed in Supplementary Appendix S3.

The obtained variable was then discretised into four modalities: “no recourse during the month,” “one recourse during the month,” “two recourses during the month” and “more than two recourses during the month.” Pairwise distances between the different patterns were then computed by Optimal Matching. Once obtained, an Ascendant Hierarchical Clustering with a Ward aggregation criterion was implemented from these distances to create care consumption clusters.

#### Main Exposure and Covariates

To test for socio-economic disparities in the DTC management course and costs, the French version of the European Ecological Deprivation Index (EDI) was used as proxy of the socio-economic position. In France, the regrouped statistical information blocks (IRIS) created by the National Institute for Statistics and Economic Studies represent the smallest geographical census unit available [13]. IRIS can be used to build the French EDI which is used as a proxy of the individual deprivation measure [14]. The EDI was discretised according to the quintiles of the French distribution. Higher modalities indicate higher levels of deprivation.

The Charlson Comorbidity Index was used to measure the comorbidity level of DTC patients included in this study. This index is derived from the FNHI database using the procedure described by Bannay et al. [15].

Based on postoperative diagnosis, an initial risk of relapse stratification was implemented, following ATA guidelines. It categorises patients into three thyroid cancer relapse risk levels: low, intermediate, and high.

#### Statistical Analyses

Quantitative variables were expressed as a mean, minimum, maximum, and standard deviation while categorical variables were expressed as a count and percentage.

To assess the impact of various characteristics on cluster distribution, a multinomial logistic regression model was employed. The care consumption patterns served as the dependent variable, and covariates included the American Thyroid Association (ATA) risk of relapse, sex, age, the European Ecological Deprivation Index (EDI), and the Charlson Comorbidity Index (CCI). Odds ratios (ORs), 95% confidence intervals (CIs), and *p*-values were reported for each covariate, with the medium consumption cluster as the reference group. Results were interpreted based on estimated ORs and associated CIs. Statistical significance was determined using the reported *p*-values, with a significance threshold set at 0.05. The reported 95% CIs resulted in a measure of uncertainty around the estimated ORs. Lack of statistical significance should be interpreted with caution, acknowledging potential trends or tendencies that did not reach the significance threshold.

Generalised estimating equations with an autoregressive working correlation structure were chosen as the statistical approach to model the expected general care consumption costs while accounting for a temporal correlation between observations. A gamma distribution and a log link were used, as this is particularly suitable for such positively skewed, continuous data. The results were reported as the estimated Relative Risk (RR) which allows a quantification of the relative change in the expected general care consumption costs associated with a one-unit change in each covariate, together with 95% CIs and *p*-values. Statistical significance was assessed using reported *p*-values, with a threshold of 0.05.

## Results

The detailed descriptive statistics of the cohort are presented in Table 1.

**TABLE 1:** Descriptive statistics of the EVATHYR cohort according to the American Thyroid Association stratified risk of relapse (France, 2024).

	ATA risk of relapse			
	Low (*N* = 195)	Medium (*N* = 49)	High (*N* = 10)	Total (*N* = 254)
Age				
Mean (SD)	52.51 (12.70)	51.94 (17.17)	43.30 (13.60)	52.04 (13.76)
Min-Max	23–86	22–83	26–75	22–86
Sex				
Women	146 (74.9%)	35 (71.4%)	9 (90.0%)	190 (74.8%)
Men	49 (25.1%)	14 (28.6%)	1 (10.0%)	64 (25.2%)
EDI				
1	47 (24.1%)	10 (20.4%)	3 (30.0%)	60 (23.6%)
2	38 (19.5%)	8 (16.3%)	1 (10.0%)	47 (18.5%)
3	41 (21.0%)	14 (28.6%)	4 (40.0%)	59 (23.2%)
4	48 (24.6%)	9 (18.4%)	1 (10.0%)	58 (22.8%)
5	21 (10.8%)	8 (16.3%)	1 (10.0%)	30 (11.8%)
CCI				
1	174 (89.2%)	45 (91.8%)	9 (90.0%)	228 (89.8%)
2	13 (6.7%)	2 (4.1%)	1 (10.0%)	16 (6.3%)
3	1 (0.5%)	1 (2.0%)	0 (0.0%)	2 (0.8%)
4	7 (3.6%)	1 (2.0%)	0 (0.0%)	8 (3.1%)

ATA stands for American Thyroid Association; SD for Standard Deviation; EDI for European Ecological Deprivation Index; CCI for Charlson Comorbidity Index.

### Description of the Different Care Consumption Patterns

**FIGURE 1:**
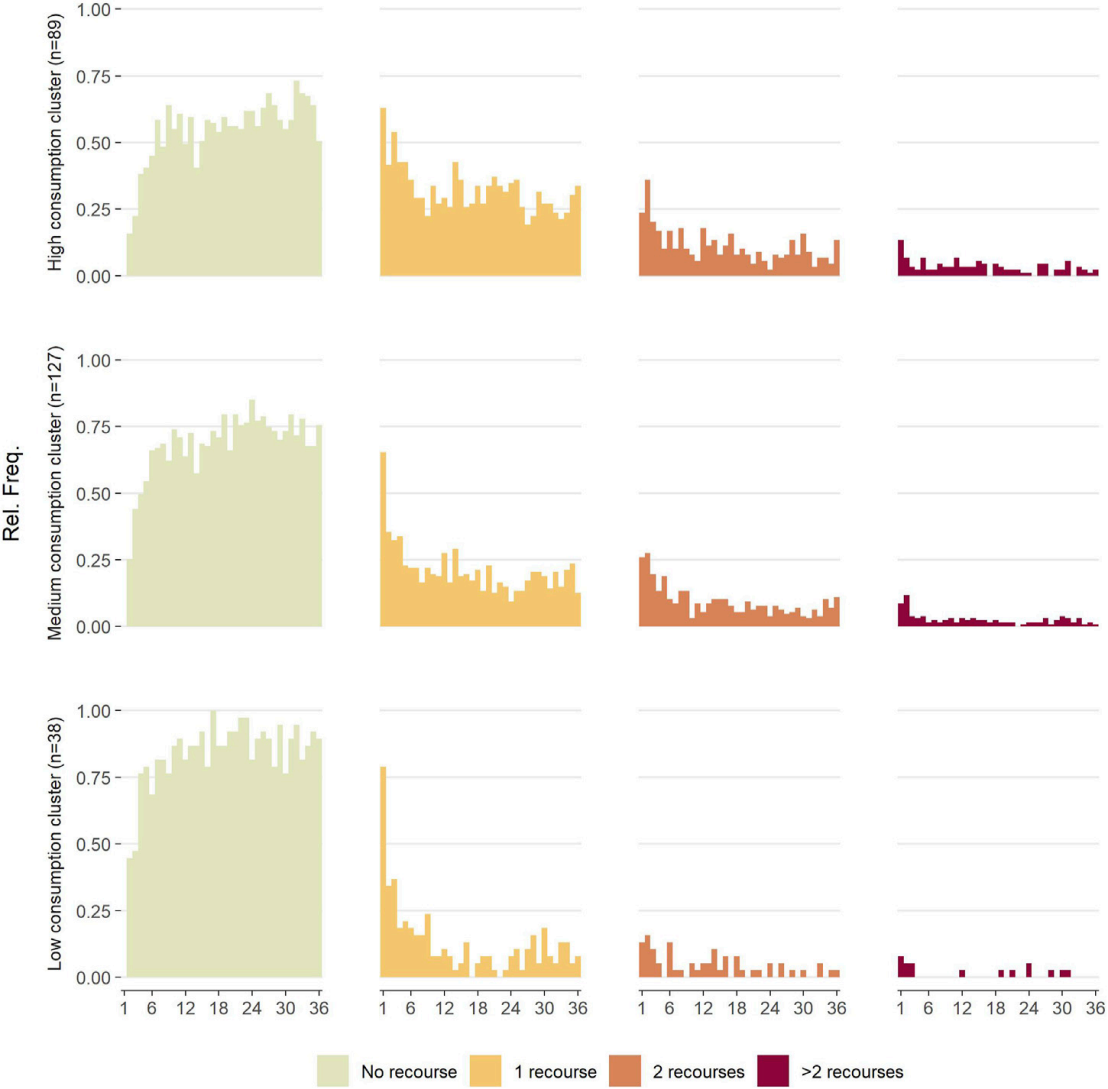
Three-year evolution of care consumption inside each cluster (France, 2024).

Sequence analysis coupled with hierarchical clustering led to the construction of a dendrogram suggesting an ideal classification in either two or three clusters. Based on the interpretability of both choices, a classification in three patterns, represented as index plots in Figure 1, was obtained. We can observe that the monthly proportions of patients using at least two services seem similar between the first cluster (*n* = 89, named “High consumption”) and the second (*n* = 127, named “Medium consumption”), but that the monthly proportions of patients using a single service are generally higher in the “High consumption” cluster. These proportions are much lower in the third cluster (*n* = 38, named “Low consumption”). Finally, in all the clusters, the consumption of care seems to decrease over time.

### Analysis of the Different Patterns

The results of multinomial logistical regression to analyse the impact of the risk of relapse following ATA guidelines and the individual characteristics on the distribution of patients within the different clusters are presented in Table 2. Although these observations do not reach statistical significance, we note trends suggesting that patients with a high risk of recurrence tend to be more likely than those with a low risk of recurrence to belong to the “high consumption” cluster rather than the “medium consumption” cluster (OR = 4.97; *p* = 0.06). Similarly, women exhibit a tendency to be more likely than men to belong to the “high consumption” cluster rather than the “medium consumption” cluster, although this trend does not achieve statistical significance (OR = 2.00; *p* = 0.05). Finally, each increase of one unit in the EDI scale increases by 26% the chances of belonging to the “high consumption” cluster rather than the “medium consumption” cluster (OR = 1.26; *p* = 0.03).

**TABLE 2:** Multivariate modelling of the impact of characteristics on the distribution within the clusters by multinomial logistic regression (France, 2024).

Covariate	High consumption cluster			Low consumption cluster		
	OR	95% CI	*p*-value	OR	95% CI	*p*-value
ATA risk of relapse						
Low	1			1		
Medium	1.55	[0.77–3.09]	0.2	0.54	[0.17–1.69]	0.3
High	4.97	[0.94–26.2]	0.06	3.54	[0.47–27.0]	0.2
Sex						
Men	1			1		
Women	2.00	[0.99–4.05]	0.05	0.96	[0.42–2.21]	>0.9
Age	1.00	[0.98–1.02]	>0.9	1.01	[0.98–1.04]	0.4
EDI	1.26	[1.02–1.55]	0.03	1.16	[0.88–1.53]	0.3
CCI						
1	1			1		
2	2.62	[0.78–8.84]	0.12	1.68	[0.37–7.64]	0.5
3	0.94	[0.06–15.8]	>0.9	0.00	[0.00–0.00]	<0.001
4	1.30	[0.28–6.04]	0.7	0.00	[0.00–0.00]	<0.001

ATA stands for American Thyroid Association; OR for Odds Ratio; CI for Confidence Interval; EDI for European Ecological Deprivation Index; CCI for Charlson Comorbidity Index. Results are relative to a reference group which is the medium consumption cluster.

### Evaluation of Three-Year Care Costs

**FIGURE 2:**
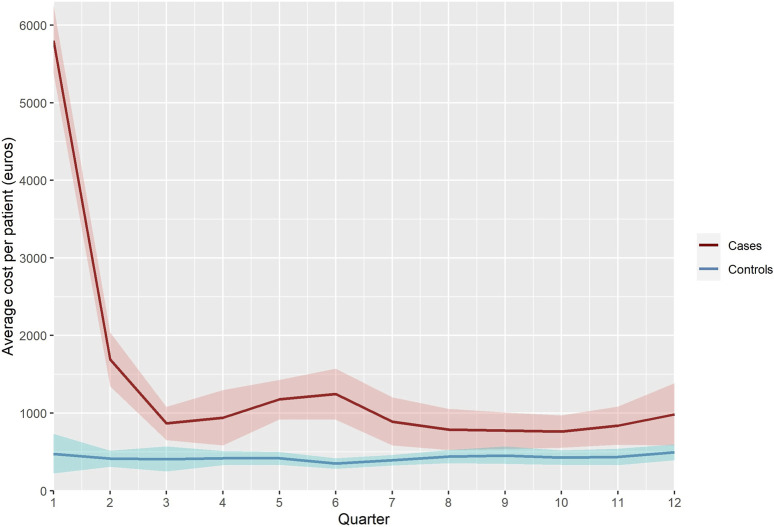
Quarterly evolution of average costs per patient, all expenditure combined (in euros 2024) (France, 2024).

**FIGURE 3:**
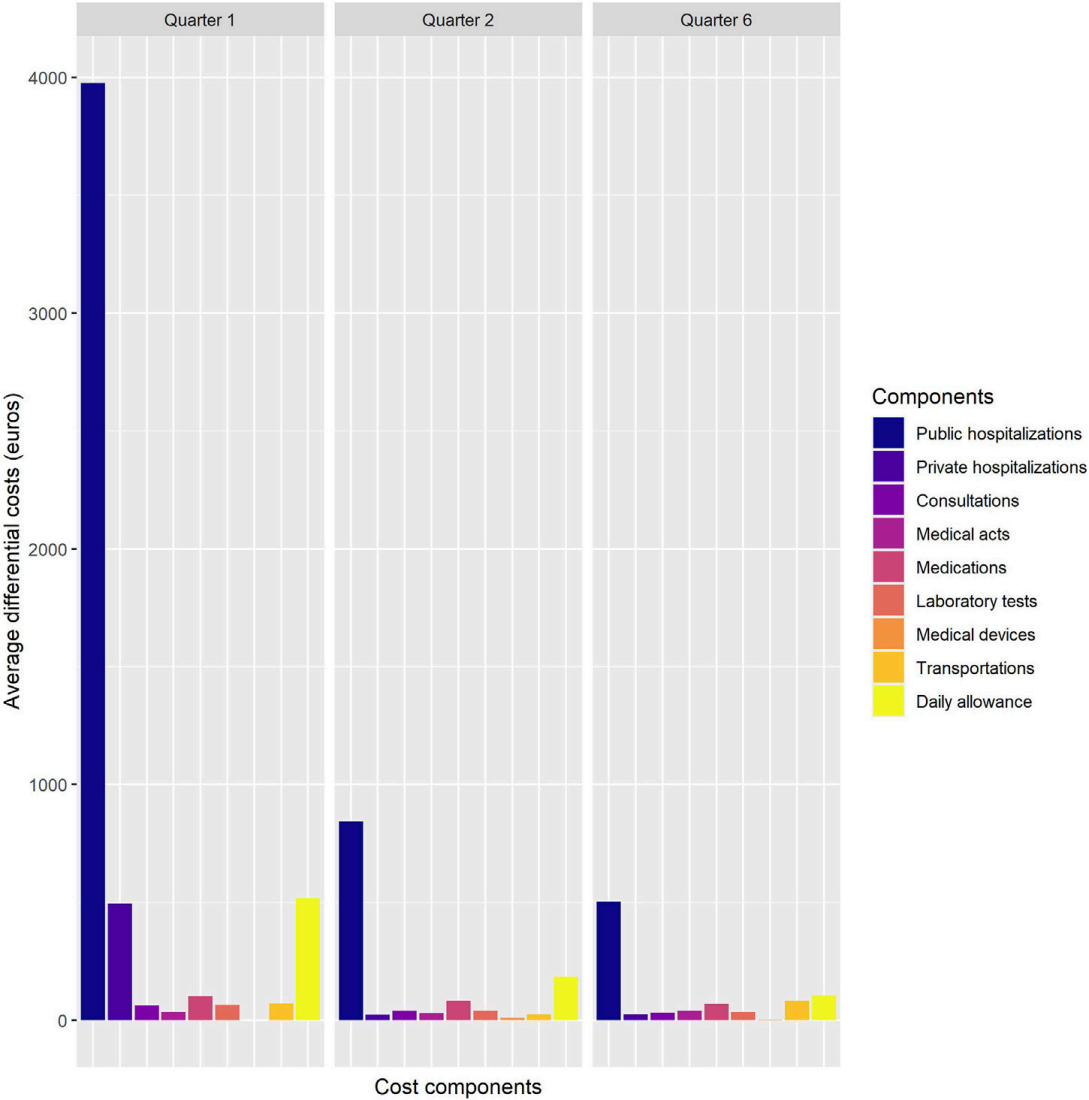
Average incremental costs by expenditure item in the first, second, and sixth quarter (in euros 2024) (France, 2024).

Figure 2 represents the quarterly evolution of average gross costs for cases and controls over a 3-year time horizon. The average quarterly cost of controls remains relatively stable and always lower than that of cases throughout the study period. Regarding cases, we observe a peak of €6,876 in the first quarter. This cost remains high in the second quarter (€2,008) and we also observe another increase during the sixth quarter (€1,476). To explain these cost increases, we analysed the various expenditure items responsible for the additional cost (Figure 3). The main expenditure items responsible for the additional cost in the first, second, and sixth trimesters were hospital stays, particularly in public establishments, and daily allowances. In the first and second trimesters, the most significant part of additional hospital costs was due to the RAI completing total thyroidectomies and surgical totalisations, as graphically represented in Supplementary Appendix S4. Finally, post-intervention thyroid check-ups represent a significant part of the cost in the sixth trimester. Overall average net costs accounted to €9,000 over the first year of follow-up, and to €13,753 over 3 years of follow-up.

### Impact of Care Consumption Patterns and Individual Characteristics on Costs

The results of the multivariate modelling of costs excluding DTC management specific care, by GEE, are presented in Table 3. We observe a significant effect of care consumption patterns on cost. In particular, patients in the “high consumption” group have a higher cost than those in the “medium consumption” group (RR = 1.35; *p* = 0.046). We also note that costs are relatively stable over time (RR = 0.95; *p* < 0.001). However, we do not observe any effect of the level of risk of recurrence on the cost of treatment. Finally, costs also increase with the number of comorbidities.

**TABLE 3:** Multivariate modelling of general care consumption costs, excluding Differentiated Thyroid Cancer management specific cares, by gamma regression (France, 2024).

Covariate	RR	95% CI	*p*-value
Care cluster			
High consumption	1.35	[1.01–1.81]	**0.046**
Medium consumption	1		
Low consumption	1.08	[0.62–1.86]	0.8
Time	0.95	[0.93–0.98]	**<0.001**
EDI	1.03	[0.90–1.17]	0.7
Age	1.01	[1.00–1.02]	0.059
ATA risk of relapse			
Low	1		
Medium	1.07	[0.80–1.43]	0.6
High	1.17	[0.56–2.44]	0.7
Sex			
Men	1		
Women	1.15	[0.87–1.53]	0.3
CCI			
1	1		
2	1.65	[1.13–2.42]	**0.01**
3	0.94	[0.60–1.48]	0.8
4	7.80	[2.70–22.5]	**<0.001**

ATA stands for American Thyroid Association; RR for Relative Risk; CI for Confidence Interval; EDI for European Deprivation Index; CCI for Charlson Comorbidity Index. Bold values were used to quickly identify which results were statistically significant (p-values < 0.05).

## Discussion

This study highlights several findings that must be interpreted, discussed, and put into perspective.

First, the analysis of global expenditure including specific healthcare services for DTC management revealed that the cost of DTC cases was consistently higher than that of controls during the study period. Peaks in expenses were observed in the first, second, and sixth quarters, and were predominantly due to hospitalisation for thyroidectomy and RAI in both public and private facilities. However, despite remaining higher, the total cost of thyroid cancer management tended to approach that of controls and to stabilise over time. Indeed, in the absence of recurrence, long-term thyroid cancer management primarily involves regular follow-up with spacing intervals and hormone therapy, which becomes episodic and relatively inexpensive. The average differential cost per patient over 3 years of follow-up was €13,753, which is about twice as much as the findings highlighted by Li et al. [6] (€7,351). This can be explained by the fact that we did not limit ourselves only to costs incurred by the management itself. In particular, Li et al. did not consider daily allowances due to absence from the workplace. Another comparison can be made with the study by Boltz et al. [4], in which they evaluated the cost of managing differentiated thyroid cancer patients aged over 65 years, from the perspective of Medicare and using data from the Surveillance, Epidemiology, and End Results cohort [16]. They found an average cost of $17,669 (€21,218) during the first year of treatment, which is significantly higher than the €9,000 we found. A first reason that could explain such a difference is that Boltz et al. focused on an elderly population, which generally incurs higher care costs. Also, notable differences between the French and American healthcare systems may account for a significant portion of this disparity. Indeed, a study by Finnerty et al. [5] assessed and compared the cost of thyroid cancer management in two samples of 100 patients each, one treated in France and the other in the United States. They reported a mean cost of $4,590 (€4,996) for the first year of treatment in France, which is relatively close to our findings considering they did not account for daily allowances, and a cost of $14,069 (€15,311) for the first year of treatment in the United States. The authors identified several key factors explaining this cost difference. Particularly, they highlighted that 19% of the cost difference could be attributed to nuclear medicine, due to Thyrogen reimbursement. Indeed, in the US, drug pricing is determined freely, whereas in the French system, pricing is the result of tripartite negotiations between pharmaceutical companies, health insurance providers, and the government. Overall, our findings differed significantly from those presented in the literature. These discrepancies can be largely attributed to the evaluation methods and the context in which the study took place. The location of care, particularly the distinct French and US healthcare systems, along with the characteristics of the studied population, such as the type of thyroid cancer and the age of individuals in the sample, contributed to these differences. Another probable source of variability may arise from the care itself, which is influenced by the unique characteristics of each patient. This is why we decided to further investigate patient care consumption patterns and their impact on care costs.

Optimal Matching is an approach that focuses on the comprehensive analysis of sequences, allowing for an examination of entire patterns, considering all relevant elements. One interesting characteristic of this approach is its ability, when paired with clustering methods, to summarise longitudinal information such as care consumption sequences into a one-dimensional variable, hence reducing its complexity into a single indicator. By synthesising the different stages, treatments, and medical decisions made throughout the trajectory into a single measure, it facilitates the analysis and comparison of different patterns. In particular, once care consumption clusters are constructed, it becomes relatively straightforward to examine their determinants, which can help us understand the factors involved in medical decision-making and contribute to improving patient care. Specifically, our research revealed that an increase in deprivation score, as measured by the EDI, tends to increase the likelihood of being a heavy consumer of specific care related to DTC management. This result differs from the findings of Zevallos et al. [7], who conducted a study using the SEER database to analyse the impact of socioeconomic position on the use of adjuvant RAI. Indeed, this study showed that patients with a lower socioeconomic position tended to be diagnosed at a more advanced stage of papillary thyroid cancer but also had a lower rate of adjuvant RAI utilisation. While healthcare costs in France are covered by the FNHI, particularly for specific cancer treatments that are fully reimbursed under the Long-Term Condition framework, in the US medical expenses are generally borne by patients, and insurance coverage is predominantly private. In such a healthcare system, it is logical that the most disadvantaged patients encounter difficulties in financing these treatments and are therefore more likely to forego additional interventions or consultations. Another conclusion highlighted by our study is that women tend, though not significantly, to have a higher likelihood of belonging to the “high consumption” cluster compared to men. This finding is consistent with the results of several studies [17–19] demonstrating that women more frequently utilise healthcare services than men, whether in the United States, Canada, or the United Kingdom. In particular, women are more inclined to consult general practitioners or specialists for their medical needs. As highlighted in the study by Yongying Wang et al. [19], this disparity persists even when considering the specific healthcare needs of women, such as reproductive health-related care.

By means of Optimal Matching Analysis and clustering techniques, this study hence highlights differences in the consumption of specific care for DTC patients. Based on these results, the main question raised was whether these differences resulted in an increased overall healthcare consumption beyond that specifically related to thyroid cancer management. The findings from the multivariate modelling of costs by generalised estimation equations demonstrate significant effects of care consumption patterns on costs excluding DTC management-specific care. Specifically, patients in the “high consumption” group were found to have higher costs compared to those in the “medium consumption” group, with a relative risk of 1.35 and a *p*-value of 0.046. Moreover, the costs were found to be relatively stable over time, with an RR of 0.95 and a *p*-value of <0.001. Furthermore, it is important to note that the estimation of costs was adjusted for comorbidities as well as for socio-economic position through the EDI in the analysis. Therefore, the conclusions regarding the observed cost differences among patients in the “high consumption” group cannot be solely explained by comorbidities or social disparities. Other factors related to care consumption patterns and management approaches likely contribute to the higher costs observed in this group. Hence, these findings suggest that DTC specific care consumption patterns play a significant role in the other general healthcare consumption costs.

This study still presents some limitations that should be mentioned and discussed. The first one concerns the size of the sample, and particularly the size of the population excluded from the initial cohort. Indeed, the exclusion of patients not found in the SNIIRAM-PMSI databases and of patients with too short follow-up leads to the loss of 107 observations, which represents approximately 30% of the initial sample, and therefore induces a loss of power in the statistical tests and analyses carried out. However, a comparison of the characteristics of the included population with those of the excluded population, presented in Supplementary Appendix S2, does not highlight any significant difference between the two groups. Secondly, the sequence analysis method presented and implemented in this study comes with a major drawback. As described, this approach only considers a single state per time step. This becomes problematic when analysing sequences that represent healthcare pathways, since it is common for patients to receive multiple healthcare services simultaneously. Here, we synthesised the information by creating a variable counting the number of healthcare services and grouping states into categories representing quantities of consumed services. Another applicable approach would be to retain the definition of states as detailed services but prioritise and choose which one to keep when multiple services occur at the same time step. However, both approaches suffer from the disadvantage of losing information. The first approach allows for the retention of all services but sacrifices the details, while the second approach, although preserving a certain level of detail, results in the exclusion of certain services. Other approaches have been developed, however, that could allow for the treatment of multidimensional sequences and the consideration of different aspects of the sequence in the analysis such as multichannel sequence analysis [20, 21]. Additionally, it must be noted that a monthly timestep was chosen for defining care consumption sequences to capture a more detailed and nuanced view of patients’ healthcare utilisation patterns trying to mitigate the issue of care simultaneity. On the other hand, a quarterly breakdown was used in the cost evaluation to strike a balance between granularity and practicality, facilitating data processing, computation times, and interpretability. Quarterly breakdowns provided a compromise between the detailed insights offered by monthly breakdowns and the simplicity associated with larger time intervals. However, as the care consumption sequences are then transformed into a qualitative variable through clustering, the impact of having used a different temporal granularity on the analysis is not expected to be tremendous. Lastly, the cost analysis was limited to a 3-year timeframe and only focused on the EVATHYR cohort. Indeed, while also confined to a 3-year assessment period, Li et al.’s study [6] relies on the General Sample of Beneficiaries, enabling them to utilise a representative sample of the French population. However, the data available in the General Sample of Beneficiaries is purely claims data. Consequently, direct access to clinical variables such as the level of cancer recurrence risk or the collection of the EDI from this sample is not feasible. To acquire these variables, it was essential to link the data from the SNIIRAM with the data from the EVATHYR cohort. In addition, to gain a more comprehensive understanding of the economic impact of DTC, it would be valuable to model the lifetime costs at a national scale in France, utilising modelling and simulation approaches. This would allow for the consideration of late recurrences and their management after RAI, which could have significant cost implications. Furthermore, as the survival of micro-papillary thyroid cancers is generally favourable, the ongoing higher consumption of general healthcare services is expected to persist for a prolonged period. Thus, evaluating the long-term additional cost associated with thyroid cancer management is crucial for informing healthcare policy and resource allocation decisions.

### Conclusion

This study reveals consistently elevated costs in thyroid cancer management, particularly during hospitalisations such as for thyroidectomies. While costs stabilise over time, variations in estimates are influenced by disparities in patient characteristics and consumption habits. Socioeconomic factors are linked to care consumption patterns, indicating higher utilisation among women—though not statistically significant—and patients facing greater deprivation.

The analysis demonstrates that patients with high consumption patterns experience significantly higher overall healthcare costs. Despite such limitations assample size constraints, the research underscores the importance of adopting a national perspective and lifetime cost modelling to comprehend the prolonged economic impact of thyroid cancer management. In essence, the study provides valuable insight into the intricate connections between care consumption patterns, costs, and individual patient characteristics in the context of thyroid cancer.
