# Supplementary material for: Identification and Economic Evaluation of Differentiated Thyroid Cancer Care Consumption Patterns Using Sequence Analysis
Source: Int J Public Health. 2024 Apr 19;69:1606664. doi: 10.3389/ijph.2024.1606664 (PMC11066236; doi:10.3389/ijph.2024.1606664)
Supplement: Supplementary file 1 [file DataSheet1.docx]

**Appendix 1. Flowchart of the EVATHYR cohort**

**
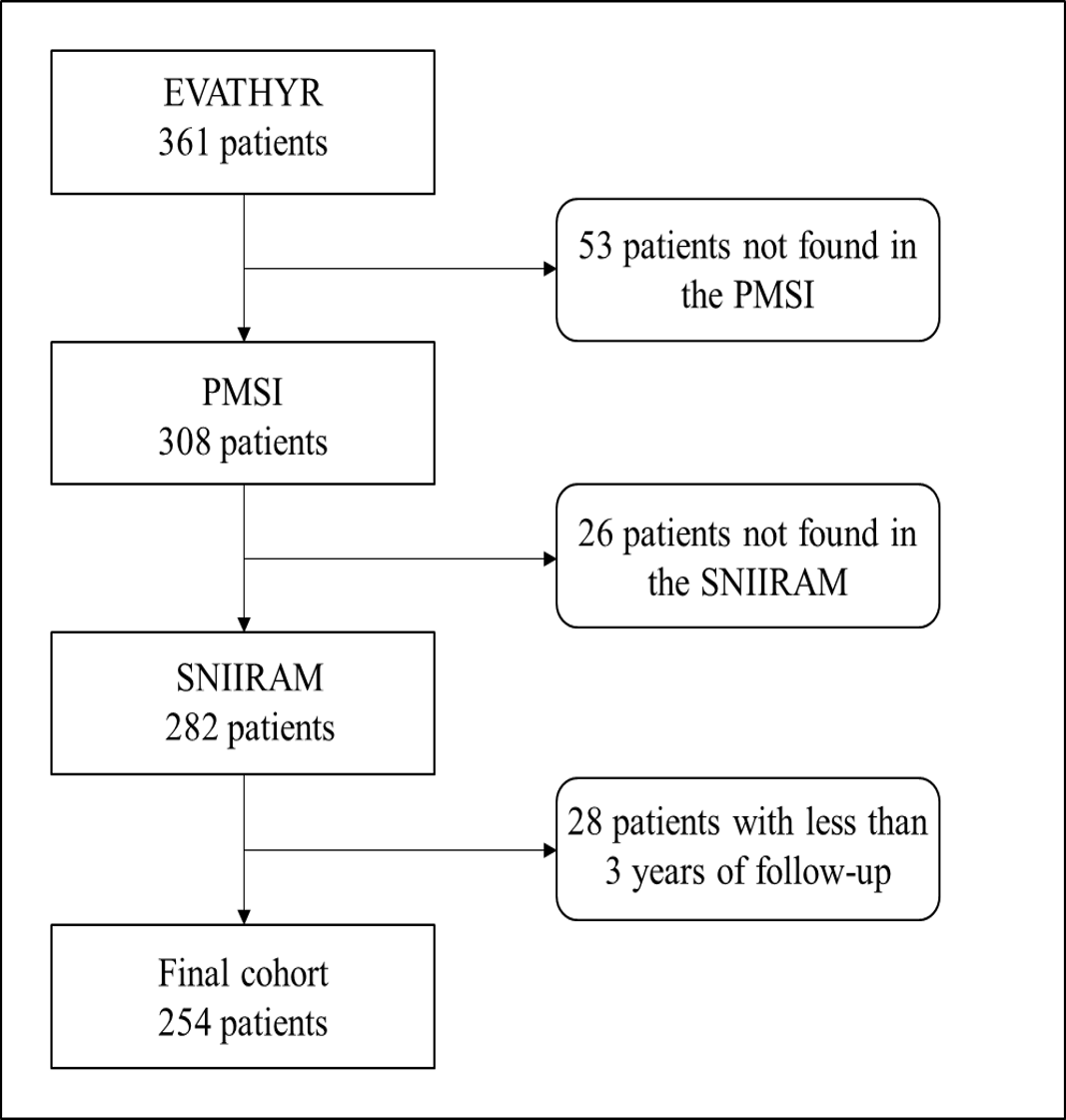
**

**Appendix 2. Baseline characteristics comparison between included and excluded patients**

|  | **Excluded (N=107)** | **Included**  **(N=254)** | **Total**  **(N=361)** | **p-value** |
| --- | --- | --- | --- | --- |
| **Age** |  |  |  | 0.181 |
| *Mean (SD)* | 54.3 (16.51) | 52.04 (13.76) | 52.71 (14.64) |  |
| *Min-Max* | 20-88 | 22-86 | 20-88 |  |
| **Sex** |  |  |  | 0.355 |
| *Women* | 75 (70.1%) | 190 (74.8%) | 265 (73.4%) |  |
| *Men* | 32 (29.9%) | 64 (25.2%) | 96 (26.6%) |  |
| **ATA risk** |  |  |  | 0.906 |
| *Low* | 83 (77.6%) | 195 (76.8%) | 278 (77.0%) |  |
| *Medium* | 19 (17.8%) | 49 (19.3%) | 68 (18.8%) |  |
| *High* | 5 (4.7%) | 10 (3.9%) | 15 (4.2%) |  |
| **EDI** |  |  |  | 0.061 |
| *1* | 21 (19.6%) | 60 (23.6%) | 81 (22.4%) |  |
| *2* | 18 (16.8%) | 47 (18.5%) | 65 (18.0%) |  |
| *3* | 21 (19.6%) | 59 (23.2%) | 80 (22.2%) |  |
| *4* | 21 (19.6%) | 58 (22.8%) | 79 (21.9%) |  |
| *5* | 26 (24.3%) | 30 (11.8%) | 56 (15.5%) |  |
| **CCI** |  |  |  | 0.093 |
| *1* | 104 (97.2%) | 228 (89.8%) | 332 (92.0%) |  |
| *2* | 3 (2.8%) | 16 (6.3%) | 19 (5.3%) |  |
| *3* | 0 (0.0%) | 2 (0.8%) | 2 (0.6%) |  |
| *4* | 0 (0.0%) | 8 (3.1%) | 8 (2.2%) |  |

ATA stands for American Thyroid Association; SD for Standard Deviation; EDI for European Deprivation Index, CCI for Charlson Comorbidity Index.

**Appendix 3. List of the DTC** **management specific cares together with their Common Classification of Medical Acts or French National Table of Biology codes.**

**Appendix 4. Incremental costs related to public hospitalizations in the first, second and sixth trimester (in euros 2014-2017)**

**
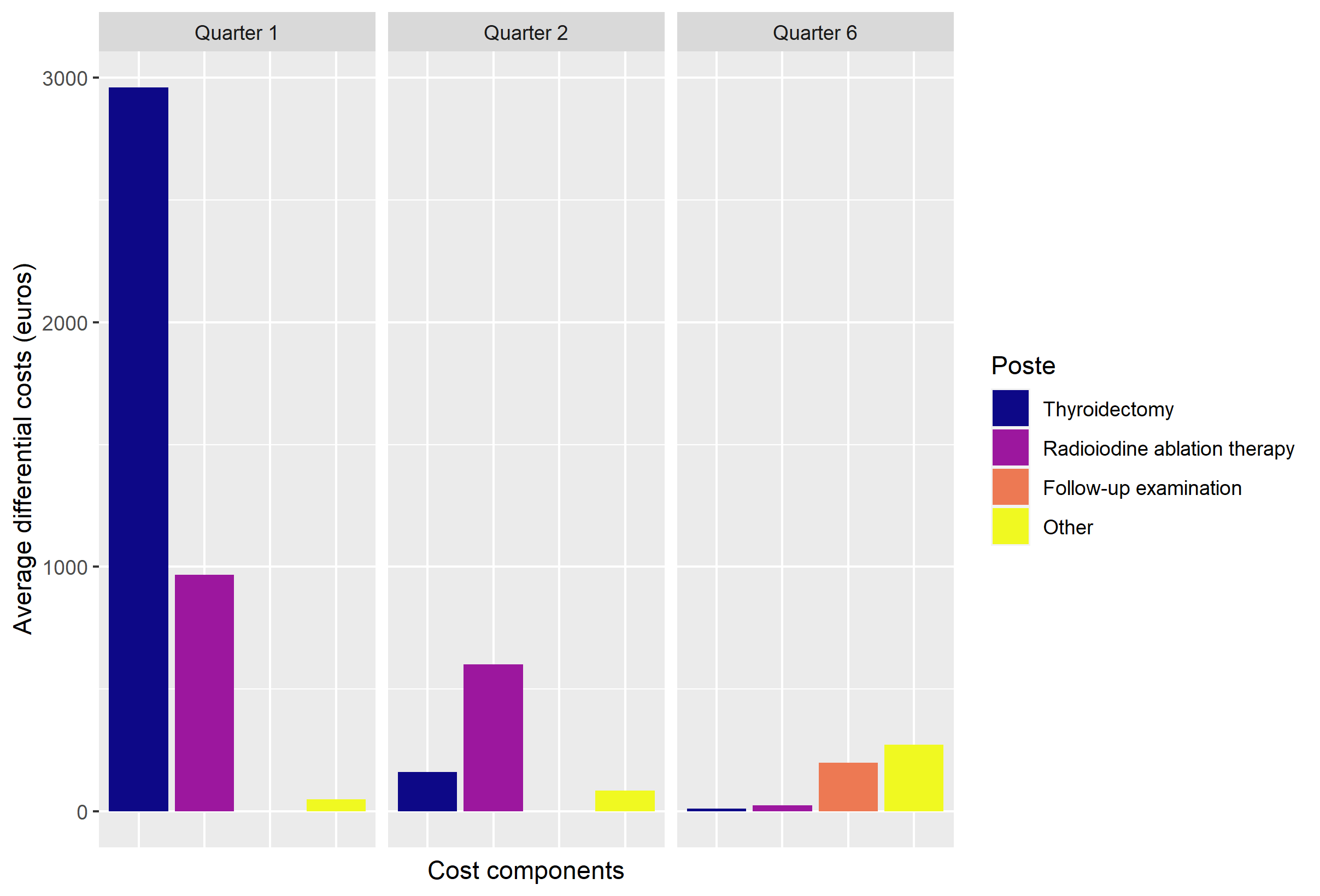
**
